# Supplementary material for: Specialised Surgical Instruments for Endoscopic and Endoscope-Assisted Neurosurgery: A Systematic Review of Safety, Efficacy and Usability
Source: Cancers (Basel). 2022 Jun 14;14(12):2931. doi: 10.3390/cancers14122931 (PMC9221041; doi:10.3390/cancers14122931)
Supplement: Supplementary file 1 [file cancers-14-02931-s001.zip › Table S1.pdf]

Supplementary Table S1: Summary of safety reporting in identified studies of available instruments for endoscopic or endoscope-assisted neurosurgery.

Instrument Name	Instrument failures/ malfunctions	Number of patients	Pathology	Procedure	Mean blood loss (ml)	Postoperative complications (number, percentage)	Intra-operative complications (number, percentage)	6-month mortality rate	Mean 6-month Glasgow Outcome Scale	General comments
2.0- μm Diode Pumped Solid State (DPSS) Laser*	0	512	Hydrocephalus, aqueductal stenosis, tumour biopsies, intracranial tumours, intracranial cysts	Rescue of ventricular catheters, endoscopic third ventriculostomy, aqeductoplasty, haemostasis, shrinkage of cyst membranes		25, 5.33%	0, 0.00%			"no long or short term morbidity and mortality"[6] "no laser related morbidity and mortality"[12]
6.3-mm Percutaneous Endoscopic Instrument	0	22	Migrated disk herniation	Transforaminal percutaneous endoscopic lumbar diskectomy		4, 18.00%	0, 0.00%			
980-nm Diode Laser	0	9	Hydrocephalus	Synechiae fenestration, endoscopic third ventriculostomy,		1, 30.00%				

				cyst wall opening					
Artemis Neuro Evacuation Device	0	1	Large suprasellar tumour with cavernous sinus invasion	Endoscopic endonasal approach	15	0, 0.00%			
Bipolar Microforceps	0	>100, 4 cases reported	Hydrocephalus, intraventricular tumour, intraventricular cysts	Endoscopic resection of tumour and fenestration					"according to our experience in more than 100 applications this bipolar instrument is safe"[16]
Bipolar Microscissors	0	100	Intracranial astrocytomas	Endoscopic surgeries	278	0, 0.00%	0, 0.00%		
BoneScalpel	0	13	Craniosynostosis	Endoscopic-assisted surgery		0, 0.00%	3, 23.00%		
Calvian Endo-Pen	0	12	Central skull base lesions	Endoscopic transsphenoidal approach					"safe and effective"[19]
Chole-Dacey Transnasal Transsphenoidal Speculum	0	90	Pituitary tumours	Endoscopic endonasal transsphenoidal approach			0, 0.00%		

EASYTRAC	0	5	Pituitary tumours	Endoscopic endonasal approach		"in no cases was there the need to remove retractor and revert to microscopic surgery"[21]
Endoscopic Curved Kerrison Rongeur	0	10	Lumbar spinal stenosis	Foramenotomy		"no cerebrospinal fluid leaks or nerve root injuries occurred"[23]
Endoscopic Stenosis Retractor	0	Cadaver	Spinal stenosis	Laminectomy		No reporting on safety
Flexible Forceps	0	20	Pituitary tumours	Endoscopic endonasal approach	0, 0.00%	
Guillotine Knife	0	34	Sylvian and suprasellar arachnoid cysts, hydrocephalus, colloid cysts	Cystocisternosty, ventriculocystocisternostomy, membrane fenestration, cystoventriculost-omy,		"all procedures were uneventful"[25]

				ventriculocystostomy, endoscopic third ventriculostomy			
Gyrus Diego Microdebrider	0	32	Skull base tumours	Endoscopic endonasal approach	1, 3.13%		"no risk of thermal injury"[26]
Haemostatic Agent Delivery	0	"variety of cases"	Bleeding from sinus and/or intraparencymal arteries	Endoscopic skull base surgery			"able to … effectively control bleeding"[27]
Handpiece for SONOCA Ultrasonic Aspirator	0	5	Pituitary adenomas, hydrocephalus	Endoscopic endonasal transsphenoidal approach, removal of intraventricular haemorrhage		0, 0.00%	
Handpiece, Keyhold, and Needle-Type Probes, and Probe Sheaths for use with the	0	119	Tumours of the brain	With operating microscope			"allowed surgeons to remove tumours rapidly, without increasing in the risk of

Ultrasonic Surgical Unit									immoderate retraction or fragmentation of surrounding normal tissue”[29]
Harmonic Scalpel	0	8	Intracrainial tumours	Endoscopic dissection of tumours					“tumours were resected efficiently and safely”[30]
HelixFlex	0	Not reported	Intracranial tumours	Endoscopic endonasal surgery					No reporting on safety
Helix Hydro-Jet	0	4	Hydrocephalus	Endoscopic third ventriculostomy			0, 0.00%		
Lotan’s Hook	0	100	Hyperhydrosis	Minimally invasive severance of thoracic sympathetic nerves		2, 2.00%	1, 1.00%		
Malleable Endoscope Suction Instrument	0	0	Haematomas	Evacuation					No reporting on safety

* = comments and outcomes from multiple studies

^ = unable to calculate percentage as number of cases states as “more than 1000”

Marburg Electrosurgical Probe, Bipolar, Flexible	0	~100	Hydrocephalus	Endoscopic third ventriculostomy, third ventriculocisternostomy					“can be safely used”[35]
Micro ENP Ultrasonic Handpiece	0	2	Intraventricular tumour	Endoscopic third ventriculostomy	1, 50%				Unclear if complication is related to instrument
Modified Flexible Grasping Forceps	0	10	Hydrocephalus	Endoscopic third ventriculostomy		0, 0.00%			
Modified Neuroendoscope Technology (MNT): a transparent sheath and haematoma smashing aspirator	0	85	Cerebral haemorrhage	Endoscopic haematoma evacuation			6.7%	3.7	
Modified Nippon Medical School Type	0	10	Sinus cavity carcinomas	Endoscopic transfacial and	0, 0.00%				

				transcortical approaches		
Modified Suction Tip	0	37	Skull base tumours	Endoscopic endonasal approach		No reporting on safety
Monopolar Suction Cautery	0	0				No reporting on safety
Monoshaft Bipolar Cautery	0	1	Left putaminal haemorrhage	Endoscopic evacuation of haematoma		"these results suggest that bipolar coagulation … facilitated secure haemostatis"[42]
NeuroBalloon	0	More than 1000	Arachnoid cysts, hydrocephalus	Endoscopic third ventriculostomy	1, ^	"except in 1 case … we have not experienced any vessel damage associated with the catheter"[43]
New Angled Chisel	0	80	Lumbar spinal stenosis	Microendoscopic decompressive laminotomy		"can be used safely even when the dura mater or nerve

							root is exposed"[44]
NICO Myriad*	1	95	Tumours of the brain and spine, aqueductal stenosis	Endoscopic endonasal, transsphenoidal, and expanded endonasal approaches, endoscopic third ventriculostomy	2, 1.05%	0, 0.00%	"we found tissue resection to be safe"[46] "no complications were directly attributable to the use of the … device"[48]
Nitinoil Stone Retrieval Basket	0	1	Colloid cyst with hydrocephalus	Endoscopic resection of colloid cyst			No reporting on safety
Novel Burr Hole Dilator	0	21	Intracranial haemorrhagic lesions	Endoscopic evacuation of haematoma via burr hole	0, 0.00%	0, 0.00%	
Novel Dilator for the Pipeline Minimally Invasive Retractor System	0	2	Parietal high grade gliomas	Coritsectomy			"the technique described … allows a safe method to perform a minimally invasive

									cortisectomy and tumour removal”[53]
Novel Rectangular Tubular Retractor	0	47	Lumbar spinal stenosis	Endoscopic bilateral decompression	51				
OmniGuide CO_2 Laser	0	16	Pituitary lesions	Endoscopic endonasal transsphenoidal approach		3, 18.75%			
Pizeoelectric System*	0	42	Craniosynostosis, pituitary adenomas, rhinogenous headache, rhinorrhea, nasal obstruction, sinusitis	Endoscopic-assisted surgery, endoscopic transnasal approach, osteotomy, osteoplasty		2, 5.26%	3, 7.89%		Post-operative complications not due to the system<br><br>“observed correct and quick healing”[57]
Pulse Laser-Induced Liquid Jet*	0	60	Pituitary lesions, skull base tumours	Endoscopic endonasal transsphenoidal and expanded endoscopic			2, 4.35%		“patients in this clinical series showed significant decreases in operative blood

				endonasal approaches	loss … compared to the operation which was performed … in our department before the introduction of this system (data not shown)”[59]
Self-retaining retractor	0	3	Pituitary adenomas	Endoscopic endonasal transsphenoidal approach	“prevents … complications by elevating the diaphragma sellae … may also increase the safety of this approach”[60]
Series of Tipped Instruments: ring curettes, dissectors, hooks, pimer	0	> 40			No reporting on safety

SONOCA Ultrasonic Aspirator*	0	21	Supratentorial intraventricular tumours	Endoscopic resection of ventricular tumours		4, 44.44%	2, 22.22%		Allowed “safer navigation and tumour resection”[62]
Sonopet Ultrasonic Bone Aspirator*	0	696	Pituitary and skull base tumours, Cervical spondylosis, ossifcation of the posterior longitudinal ligament, cervical diseases, thoracic ossification, lumbar canal stenosis, tumours, Chiari malformation	Endoscopic endonasal transsphenoidal and endoscopic endonasal approaches, spinal surgeries	16.5<br>5	0, 0.00%	6, 0.86%		“all dural damage occurred in cases in which the edge of the bone harbouring dura mater was removed”[65]<br><br>“all surgeons agreed that the UA increased safety”[66]<br><br>“no heat or mechanical injury was observed”[67]
Suction Device made of Shape Memory Alloy	0	0							No reporting on safety

connected to ATOM5 Record 55 DDS							
Trapezoidal Specula	0	Model	Pituitary tumours	Endoscopic transsphenoidal approach			No reporting on safety
Ultrasonic Aspirator Tube	0	8	Intraventricular haemorrhage, acute hydrocephalus	Neuroendoscopic evacuation	0, 0.00%		"all patients tolerated procedure well"[70]
XS Micro Instruments	0	0					No reporting on safety
ZESSYS	0	70	Lumbar disc herniation	Percutaneous endoscopic lumbar disectomy	0, 0.00&		"Reduced radiation exposure time"[72]
