# Supplementary material for: Specialised Surgical Instruments for Endoscopic and Endoscope-Assisted Neurosurgery: A Systematic Review of Safety, Efficacy and Usability
Source: Cancers (Basel). 2022 Jun 14;14(12):2931. doi: 10.3390/cancers14122931 (PMC9221041; doi:10.3390/cancers14122931)
Supplement: Supplementary file 1 [file cancers-14-02931-s001.zip › Table S2.pdf]

Supplementary Table S2: *Summary of operative outcome reporting in identified studies of available instruments for endoscopic or endoscope-assisted neurosurgery.*

Instrument Name	Number of patients	Pathology	Procedure	Gross total resection (number, percentage)	Subtotal resection (number, percentage)	Partial resection (number, percentage)	Mean operative time (mins)	General comments
2.0- μm Diode Pumped Solid State (DPSS) Laser*	512	Hydrocephalus, aqueductal stenosis, tumour biopsies, intracranial tumours, intracranial cysts	Rescue of ventricular catheters, ETV, aqeductoplasty, haemostasis, shrinkage of cyst membranes				72<br>99	
6.3-mm Percutaneous Endoscopic Instrument	22	Migrated disk herniation	Transforaminal percutaneous endoscopic lumbar diskectomy					"The new instrument was useful in 13 of 22 patients, even outside the resection range while using conventional forceps."[13]
980-nm Diode Laser	9	Hydrocephalus	Synechiae fenestration, endoscopic third ventriculostomy, cyst wall opening					"a versatile tool for a broad range of applications"[14]

Artemis Neuro Evacuation Device	1	Large suprasellar tumour with cavernous sinus invasion	Endoscopic endonasal approach	<10	
Bipolar Microforceps	>100, 4 cases reported	Hydrocephalus, intraventricular tumour, intraventricular cysts	Endoscopic resection of tumour and fenestration		"according our experience in more than 100 applications … this bipolar instrument is safe and accurate"[16]
Bipolar Microscissors	100	Intracranial astrocytomas	Endoscopic surgeries	114	
BoneScalpel	13	Craniosynostosis	Endoscopic-assisted surgery	84	
Calvian Endo-Pen	12	Central skull base lesions	Endoscopic transsphenoidal approach		"effective during EETS for central skull base pathologies"[19]
Chole-Dacey Transnasal Transsphenoidal Speculum	90	Pituitary tumours	Endoscopic endonasal transsphenoidal approach		No reporting on efficacy
EASYTRAC	5	Pituitary tumours	Endoscopic endonasal approach	48	

Endoscopic Curved Kerrison Rongeur	10	Lumbar spinal stenosis	Foramenotomy	"allowed the ligamentum to be removed … much more easily than by using an operative microscope or magnifying loops"[23]
Endoscopic Stenosis Retractor	Cadaver	Spinal stenosis	Laminectomy	"retractor was successful at monitoring the use of angled curettes and punches within the lateral recess"[22]
Flexible Forceps	20	Pituitary tumours	Endoscopic endonasal approach	"able to access sites where regular dedicated instruments … could not easily reach"[19] "facilitated better manipulation … compared with

								standard dedicated instruments”[24]
Guillotine Knife	34	Sylvian and suprasellar arachnoid cysts, hydrocephalus, colloid cysts	Cystocisternostomy, ventriculocystocisternostomy, membrane fenestration, cystoventriculostomy, ventriculocystostomy, endoscopic third ventriculostomy					“all procedures were … technically successful”[25]
Gyrus Diego Microdebrider	32	Skull base tumours	Endoscopic endonasal approach	26, 81.30%	4, 12.50%	2, 6.30%		
Haemostatic Agent Delivery	“variety of cases”	Bleeding from sinus and/or intraparencymal arteries	Endoscopic skull base surgery					No reporting on efficacy
Handpiece for SONOCA Ultrasonic Aspirator	5	Pituitary adenomas, hydrocephalus	Endoscopic endonasal transsphenoidal approach, removal of intraventricular haemorrhage					“efficacy found to be good with reliable tissue aspiration”[28]
Handpiece, keyhold and needle-type probes, and probe sheaths	5	Pituitary adenomas, hydrocephalus	Endoscopic endonasal transsphenoidal approach, removal of intraventricular haemorrhage					"the keyhold-type probes could reach all regions, and they functioned efficiently"[29]

for use with the Ultrasonic Surgical Unit					
Harmonic Scalpel	8	Intracrainial tumours	Endoscopic dissection of tumours		“tumours were resected efficiently”[30]
HelixFlex	Not reported	Intracranial tumours	Endoscopic endonasal surgery		No reporting on efficacy
Helix Hydro-Jet	4	Hydrocephalus	Endoscopic third ventriculostomy		“precise and accurate perforation”[32]
Lotan’s Hook	100	Hyperhydrosis	Minimally invasive severance of thoracic sympathetic nerves	15<br>40	“there are significant advantages to operating with the Lotan hook”[33]
Malleable Endoscope Suction Instrument	0	Haematomas	Evacuation		“allows visualisation of the working channel … not as large or bulky as the flexible endoscope”[34]

Marburg Electrosurgical Probe, Bipolar, Flexible	~100	Hydrocephalus	Endoscopic third ventriculostomy, third ventriculocisternostomy			"successfully used in about 100 cases"[35]
Micro ENP Ultrasonic Handpiece	2	Intraventricular tumour	Endoscopic third ventriculostomy			No reporting on efficacy
Modified Flexible Grasping Forceps	10	Hydrocephalus	Endoscopic third ventriculostomy			No reporting on efficacy
Modified Neuroendoscope Technology (MNT): a transparent sheath and haematoma smashing aspirator	85	Cerebral haemorrhage	Endoscopic haematoma evacuation		86.8	
Modified Nippon Medical School Type	10	Sinus cavity carcinomas	Endoscopic transfacial and transcortical approaches			No reporting on efficacy
Modified Suction Tip	37	Skull base tumours	Endoscopic endonasal approach			"showed good handling of

						surgicel and gelfoam”[40]
Monopolar Suction Cautery	0					“proven to be a valid and effective instrument”[41]
Monoshaft Bipolar Cautery	1	Left putaminal haemorrhage	Endoscopic evacuation of haematoma			“can be used without difficulty”[42]
NeuroBalloon	More than 1000	Arachnoid cysts, hydrocephalus	Endoscopic third ventriculostomy			“rate of rupture of the balloon during the procedure was less than 2%”[43]
New Angled Chisel	80	Lumbar spinal stenosis	Microendoscopic decompressive laminotomy			“clinical and radiological results in the NAC group were better … the NAC sharpened the osteotomy angle and thereby improved the clinical course”[44]
NICO Myriad*	95	Tumours of the brain and spine,	Endoscopic endonasal, transsphenoidal, and expanded endonasal	21, 67.4%<br>10, 76.92%	9, 29.03%	“no residual tumour”[45]

		aqueductal stenosis	approaches, endoscopic third ventriculostomy	“easy to use and effective in the removal of lesions”[47] “efficient in debulking the firm tumour”[50]
Nitinoil Stone Retrieval Basket	1	Colloid cyst with hydrocephalus	Endoscopic resection of colloid cyst	“successfully used a flexible kidney stone basket that could be maneuvered in the ventricular system in an atraumatic fashion”[51]
Novel Burr Hole Dilator	21	Intracranial haemorrhagic lesions	Endoscopic evacuation of haematoma via burr hole	“extended burr hole in all cases”[52]
Novel Dilator for the Pipeline Minimally Invasive Retractor System	2	Parietal high grade gliomas	Cortisectomy	“using … the described dilator decreases the cortisectomy size, reduces brain trauma, and allows an effective

								method of tumour removal"[53]
Novel Rectangular Tubular Retractor	57	Lumbar spinal stenosis	Endoscopic bilateral decompression				62	
OmniGuide CO_2 Laser	16	Pituitary lesions	Endoscopic endonasal transsphenoidal approach	7, 43.75%	1, 6.25%	2, 12.50%		
Pizeoelectric System*	42	Craniosynostosis, pituitary adenomas, rhinogenous headache, rhinorrhea, nasal obstruction, sinusitis	Endoscopic-assisted surgery, endoscopic transnasal approach, osteotomy, osteoplasty				84<br>90	"the advantage of the piezoelectric is the precise and selected cutting on bone and cartilage"[57]
Pulse Laser-Induced Liquid Jet*	60	Pituitary lesions, skull base tumours	Endoscopic endonasal transsphenoidal and expanded endoscopic endonasal approaches	39, 83.78%<br>6, 50.00%	1, 8.33%	7, 15.22%<br>5, 41.66%		"satisfactory accessibility in all cases"[59]
Self-Retaining Retractor	3	Pituitary adenomas	Endoscopic endonasal transsphenoidal approach	3, 100.00%				"permits bimanual work"[60]
Series of Tipped Instruments: ring curettes,	> 40							"applied in over 40 procedures"[61]

dissectors, hooks, pimer								
SONOCA Ultrasonic Aspirator*	21	Supratentorial intraventricular tumours	Endoscopic resection of ventricular tumours	9, 42.85%	7, 33.33%	5, 23.81%	70	"in all cases biopsy was possible"[63]
Sonopet Ultrasonic Bone Aspirator*	696	Pituitary and skull base tumours, Cervical spondylosis, ossifcation of the posterior longitudinal ligament, cervical diseases, thoracic ossification, lumbar canal stenosis, tumours, Chiari malformation	Endoscopic endonasal transsphenoidal and endoscopic endonasal approaches, spinal surgeries	10, 1.43%			31.92	"allowed straight visual access … allows for both irrigation and aspiration … highly useful in various surgical procedures"[65] "well suited for transnasal approaches in skull base surgery … the surgical goals could be achieved"[66]
Suction Device made of Shape Memory Alloy connected to	0							"surgeons rated the ability to bend the suction device in every desired

ATOM5 Record 55 DDS						form as very good”[68]
Trapezoidal Specula	Model	Pituitary tumours	Endoscopic transsphenoidal approach			“shorter specula yield wider angles of exposure in the horizontal plane … and improves instrument maneuverability”[69]
Ultrasonic aspirator tube	8	Intraventricular haemorrhage, acute hydrocephalus	Neuroendoscopic evacuation	8, 100.00%		
XS Micro Instruments	0					No reporting on efficacy
ZESSYS	70	Lumbar disc herniation	Percutaneous endoscopic lumbar disectomy		44.51	

* = comments and outcomes from multiple studies
