# Supplementary material for: Specialised Surgical Instruments for Endoscopic and Endoscope-Assisted Neurosurgery: A Systematic Review of Safety, Efficacy and Usability
Source: Cancers (Basel). 2022 Jun 14;14(12):2931. doi: 10.3390/cancers14122931 (PMC9221041; doi:10.3390/cancers14122931)
Supplement: Supplementary file 1 [file cancers-14-02931-s001.zip › Table S3.pdf]

Supplementary Table S3: *Summary of reporting to ergonomic and learning curve assessment in identified studies of available instruments for endoscopic or endoscope-assisted neurosurgery.*

Instrument Name	Comment on ergonomic assessment	Comment on learning curve
2.0- μm Diode Pumped Solid State (DPSS) Laser		"has a learning curve regarding the reaction of different types of tissue"[12]<br>"learning curve was experienced by all surgeons as steep and quick"[12]
Bipolar Microscissors	"functional combination of two instruments did not limit freedom of surgical manoeuvres"[17]	"no special training is needed to use the tool, just a little familiarity with it"[17]
Haemostatic Agent Delivery	"entire process can be performed using only one hand"[27]	
Handpiece, Keyhold and Needle-Type Probes, and Probe Sheaths for use with the Ultrasonic Surgical Unit	"The light weight of the handpiece rendered it stable and easy to handle."[29]	
Malleable Endoscope Suction Instrument	"light and easy to manipulate"[34]	
Monoshaft Bipolar Cautery	"a single-handed method makes it possible to gain some mobility with both the endoscope and the monoshaft bipolar, thus making it possible to perform more than one task with a single hand"[42]	
NeuroBalloon		"the use of the NeuroBalloon catheter in our experience may shorten this learning process by facilitating the dilation of the stoma in the floor of the third ventricle"[43]
NICO Myriad		"disadvantages include … learning curve associated with the device (as with any device)"[46]

SONOCA Ultrasonic Aspirator		"purely single-portal endoscopic resection of intraventricular tumours is complex and requires a specific technique with its associated learning curve"[63]
Sonopet Ultrasonic Bone Aspirator*	"The handling and operating of the UA was described as intuitive and easy, and additional and physical or cognitive workload was considered minimal."[66]	"there did not appear to be a difference in learning curve … primary surgeon had used this instrument in about 7 cases prior to the start of this study, and there was no objective decrease in operative time or subjective difference in ease of use"[44]<br><br>"The application of the new device was easily learned."[66]<br><br>"The ultrasonic bone curette does not require a significant learning curve."[67]
Suction Device made of Shape Memory Alloy connected to ATOM5 Record 55 DDS	"the ergonomics of the type 4 handpiece were rated as *very good*, and this type now serves as the primary design"[68]	
ZESSYS		TESSYS (comparison instrument) has "steep learning curve", whereas the ZESSYS "had significant advantages over the traditional TESSYS foraminoplasty technique". The ZESSYS "could reduce the technique difficulties" and "for a junior surgeon, these differences would be more obvious."[72]

*complied from multiple studies
