# Supplementary material for: Specialised Surgical Instruments for Endoscopic and Endoscope-Assisted Neurosurgery: A Systematic Review of Safety, Efficacy and Usability
Source: Cancers (Basel). 2022 Jun 14;14(12):2931. doi: 10.3390/cancers14122931 (PMC9221041; doi:10.3390/cancers14122931)
Supplement: Supplementary file 1 [file cancers-14-02931-s001.zip › Table S4.pdf]

Supplementary Table S4: *Summary of studies reporting comparison to standard instruments. (Key: S = Standard Instrument, N = New Instrument)*

Instrument Name	Number of patients	Pathology	Procedure	Comparison instrument(s)	Mean blood loss (ml)		Mean ICU monitoring time (days)		6-month mortality rate		Mean 6-month Glasgow Outcome Scale		Mean operative time (mins)	
					S	N	S	N	S	N	S	N	S	N
Bipolar Microscissors	100	Intracranial astrocytomas	Endoscopic surgeries	Not stated	361	278							145	114
Flexible Forceps	20	Pituitary tumours	EEA	"dedicated instruments for excision"[24]										
Harmonic Scalpel	8	Intracranial tumours	Endoscopic dissection of tumours	Conventional devices such as monopolar and bipolar diathermy										
Modified Neuroendoscope Technology (MNT): a transparent sheath and haematoma smashing aspirator	85	Cerebral haemorrhage	Endoscopic haematoma evacuation	External drainage and monitoring system (Medtronic, USA)			16.2	5	22.5%	6.7%	2.9	3.7	39.7	86.8

New Angled Chisel	80	Lumbar spinal stenosis	Microendoscopic decompressive laminotomy	“Same procedure without the new angled chisel”[44]									
Novel Rectangular Tubular Retractor	47	Lumbar spinal stenosis	Endoscopic bilateral decompression	Cylindrical tubular retractor								71	62
Piezoelectric System	15	Pituitary adenomas	Endoscopic transnasal approach	Standard								147	90
Sonopet Ultrasonic Bone Aspirator	130	Pituitary tumours	EETSA	Kerrisons, thru-cuts, backbiters, Jansen-Middleton instrumentation	22.58	16.55						41.33	31.92
ZESSYS	70	Lumbar disc herniation	Percutaneous endoscopic lumbar disectomy	TESSYS								45.93	44.51
